# Determinants of postnatal care utilization in Tigray, Northern Ethiopia: A community based cross-sectional study

**DOI:** 10.1371/journal.pone.0221161

**Published:** 2019-08-20

**Authors:** Almaz Berhe, Alemayehu Bayray, Yibrah Berhe, Alula Teklu, Amanuel Desta, Tsige Araya, Ruth Zielinski, Lee Roosevelt

**Affiliations:** 1 Mekelle University, School of Medicine and Health Sciences, Addis Adaba, Ethiopia; 2 St. Paul’s Hospital Millennium Medical College, Addis Adaba, Ethiopia; 3 Center for International Reproductive Health Training, Ann Arbor, Michigan, United States of America; 4 University of Michigan, School of Nursing, Ann Arbor, Michigan, United States of America; Kwame Nkrumah University of Science and Technology, GHANA

## Abstract

**Introduction:**

Globally, 289,000 women die from complications related to pregnancy, childbirth, or the postnatal period every year. Two-thirds of all maternal deaths occur during the first six weeks following birth and more than two thirds of newborn deaths occur during the first week of life, These statistics underscore the importance of postnatal care, an often neglected service according to the World Health Organization (WHO). The purpose of this study was to assess the factors associated with postnatal service utilization in the Tigray region of Ethiopia.

**Methods:**

The study was a community-based, cross-sectional study. A multi-stage sampling method was used to select study districts randomly from the entire region. A total of 1,690 participants were selected using systematic random sampling. Participants were 18–49 years old, had given birth within the last six months, and were residents of the district for at least six months. Using SPSS version 20 means, frequencies, and percentages were calculated for the sub-group of participants who did attend postnatal care. Barriers to non-attendance of postatal care were analyzed using descriptive statistics. Bivariate analysis was undertaken to assess the association between demographic, obstetric, and knowledge regarding PNC and attendance at antenatal care. Variables with a P value, <0.05 were included in the multivariate logistic regression analysis to identify the determinant factors of postnatal care utilization.

**Result:**

Of the women surveyed, 132 (8%) obtained postnatal care. Women who did not receive postnatal care reported lack of awareness of the services (n = 1110, 73.3%). Most mothers who received postnatal care reported that they were aware of the service prior to the birth of their child (n = 101, 76.5%). Women were more likely to receive postnatal services if they lived in an urban area (odds ratio 1.96, 95% confidence interval 1.07, 3.59), had greater than a secondary education (OR 3.60, 95% CI 1.32,9.83), delivered by cesarean section (OR 2.88 95% CI 1.32,6.29), had four or more antenatal visits (OR 4.84, 95% CI 1.57,14.9), or had a planned pregnancy (OR 6.47, 95% CI 2.04,20.5).

**Conclusion:**

Postnatal care service utilization is very low in Tigray region. Interventions targeted at increasing women’s awareness of the importance of postnatal services and improving accessibility, particularly in rural areas, is needed.

## Introduction

The developing world has the highest prevalence of maternal and infant morbidity and mortality. Almost all (99%) of maternal and neonatal deaths occur in developing countries [1,2]. Most maternal and infant deaths occur in the first six weeks after childbirth [3, 4].

Neonates account for an increasing share of child deaths, reaching almost half (45%] of the burden of under-five mortality [3]. The postnatal period (the time just after delivery and through the first six weeks of life] is especially critical for newborns and mothers [5].

In low income countries, almost 40% of women experience complications after delivery and an estimated 15% develop potentially life-threatening problems [6]. Ethiopia, one of the countries in sub-Saharan Africa, is among the six countries that contribute to approximately 50% of the maternal deaths worldwide [7]. Ethiopia has a maternal mortality rate of 412 per 100,000 and an infant mortality rate of 48 per 1000 live births [8].

Postnatal care service is a fundamental element of the continuum of essential maternity care–which also includes antenatal care and skilled birth attendance. These three elements have been demonstrated to decrease maternal and neonatal morbidity and mortality in low- and middle-income countries, particularly when all three elements are available and utilized [9, 10]. However, the provision of skilled postnatal care is relatively poor compared with that given before and during childbirth[11]. Increasing access and utilization of postnatal care has been identified as a priority in prior research [12]. In Ethiopia, while approximately 62% of women received antenatal care from a skilled provider, only 7% received the recommended postnatal care within two days of delivery, the time identified by Safe Motherhood programs as critical for complication recognition [8].

Provision of postnatal care, using the World Health Organization (WHO) recommendations, includes four visits: within 24 hours of birth, two to three days, six to seven days, and at six weeks [11]. Low birth weight (LBW) infants, as well as HIV-positive mothers, require an extra two or three visits. Lack of appropriate care during this period could result in significant ill health and even death as most maternal and infant deaths occur during this time period [11]. Therefore, the purpose of this research project was to determine the factors associated with postnatal care utilization among women in the Tigray region of Ethiopia in an effort to inform future initiatives seeking to increase postnatal care utilization.

## Materials and methods

We defined postnatal care as a woman and newborn receiving care from a nurse, midwife, or physician. While care provision by a health extension worker does have value, these workers may not have the equipment or skill necessary to measure important factors such as blood pressure or newborn weight.

### Study setting

**Fig 1:**
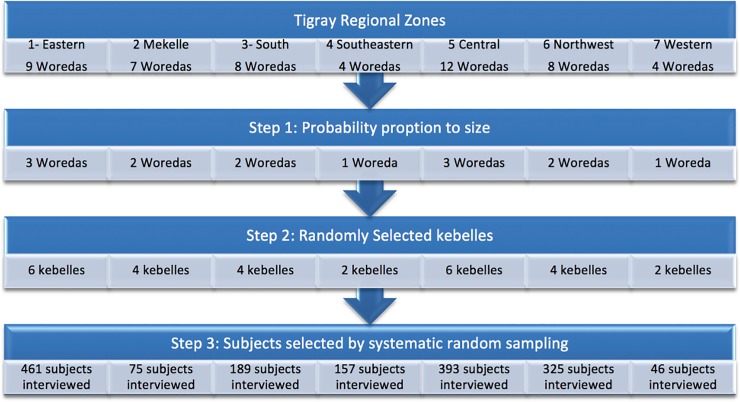
Schematic presentation of the samplicing procedure. Tigray Region is the northernmost of the nine regions of Ethiopia. There are seven administrative zones: comprising a total of 47 *Woredas* (districts) and 673 *Kebelles* (neighborhoods).

The study was conducted in the Tigray regional state located in the northern part of Ethiopia, approximately 800 km from Addis Ababa, the capital city of Ethiopia. According to the “population and housing census -2007” the region has a total population of 6,960,003[13]. It is administratively divided into seven zones, which were used for our sampling strategy (Fig 1). Each zone is comprised of Woredas, which are the third-level administrative divisions. Woredas are further subdivided into kebelle, or neighborhood associations, the smallest unit of local government in Ethiopia. The Tigray region has 52 Woredas, (34 rural and 19 urban) and 799 kebelles (722 rural and 77 urban). The health care coverage currently reaches around 83% of the population with five zonal hospitals, nine district general hospitals, two referral university hospitals, 214 health centers and 613 health posts—all run by the government [14]. There are also a number of private hospitals and clinics, but information regarding whether these hospitals and clinics provide postnatal care by a skilled professional is not readily available.

### Sampling procedure

The study employed a community based, cross-sectional study design of mothers from the ages of 18 to 49 years who gave birth in health facilities or at home in the previous six months in randomly selected kebelles from randomly selected woredas. Mothers were randomly selected for inclusion in the study. All subjects were residents of their kebelle for at least six months (Fig 1). A single proportion formula was used to identify a required sample size of 1690 assuming a 95% confidence interval, a margin of error of 4%, a population size >10,000 and a 20% prevalence of PNC [15].

First, the targeted sample number for each Woredas was determined across the seven zones by probability proportional to size (PPS) using the number of postnatal mothers (mothers delivered in the last 6 months) for each Woredas. Next, the targeted sample number was determined by PPS across each Woredas, to randomly select kebelles. Third, eligible households within each kebelle were selected by a simple, random sampling technique. To select the first eligible household in each village, the study team started at a central place in the village, spun a pen, and walked to the edge of the village in the direction that the pen pointed. A random house was selected by lottery method to identify the starting household for the cluster and collection continued on the right-hand side of this starting house until the required number of individuals had been recruited for the sample [16, 17]. Only one participant was included from each house. If no one was home, or if no eligible participants were at a house, the next closest household was taken. Eligibility of participants was confirmed by asking age, date of delivery and duration of stay in the district. A total of 68,253 households were approached from which 1,690 eligible participants were identified and 1,646 were willing to participate, making the acceptance rate 97.4%.

### Data collection and quality control

The questionnaire was developed through review of related Ethiopian and international literature [6, 8, 14, 15, and 16]. The questionnaire was prepared by the principal investigators, first in English then translated into the Tigrigna language (the local language of the area), and back to English in order to ensure its consistency. The questionnaire included questions regarding socio-economic status, demographic variables, reproductive history, maternity care, and postnatal care. The questionnaire was piloted in non-study areas of the Tigray region prior to study data collection; minor modifications such as skip pattern, ambiguity, timing and sequence were made to the questionnaire prior to the actual data collection.

### Ethical considerations

Ethical clearance for the study was obtained from the Institutional Review Board of Mekelle University and a letter of permission was obtained from the Tigray regional health bureau. Data collectors verbally explained the purpose and nature of the research and read the informed consent document aloud to all potential participants who were unable to read it independently. Consent was affirmed by participant signature or fingerprint on the consent document.

### Data analysis

The data were entered into EPI data version 3.1 and exported to SPSS version 20 for analysis. Descriptive statistics (means, standard deviations and percentages) were computed to describe the study population, including background characteristics, obstetric history, and participant knowledge of maternal and infant danger signs. For the sub-group of participants who did attend postnatal care, reasons for attending, mode of transport to the healthcare facility, and wait time were analyzed using means, frequencies, and percentages. Barriers to non-attendance at postnatal care were analyzed using descriptive statistics. Bivariate analysis was undertaken to assess the association between demographic, obstetric, and knowledge regarding PNC and attendance at antenatal care. Of these, variables with a P value, <0.05 were included in the multivariate logistic regression analysis to identify the determinant factors of postnatal care utilization.

## Results

### Socio-demographic descriptive characteristics of participants

The total 1,646 participants ranged in age from 18–49 years old, most (n = 1,320, 80.2%), were 20–34 years old. The mean age of respondents was 27.3 years (standard deviation SD ± 5.8.0). More than half of the participants (62.1%, n = 1022) earned less than 1500 Ethiopian Birr (55.5 USD) per month. The mean monthly income of the respondents was 1902 Birr [~70.4 USD]. The majority of respondents (86.1%, n = 1,417) were rural dwellers and most (84.8%, n = 1,395) were married. Almost a third (31.8%, n = 524) reported no formal education, 39% (n = 642) completed primary school, 22.6% (n = 372) completed secondary school, and 6.6% (n = 108) had some post-secondary education (Table 1).

**Table 1:** Background characteristics of participants (n = 1,646).

Variable	Category	Frequency n (%)
Age	18–19 yrs.	112 (6.80)
	20–34 yrs.	1,320 (80.2)
	35–49 yrs.	214 (13.0)
Religion	Orthodox Christian	1,401 (85.1)
	Muslim	231 (14.0)
	Other(Catholic, Protestant)	14 (0.9)
Ethnicity	Tigray	1,605 (97.5)
	Amara	29 (1.8)
	Oromo	12 (0.7)
Marital status	Married	1,395 (84.8)
	Single	63 (3.8)
	Separated	108 (6.6)
	Widowed or Divorced	77 (4.8)
Woman’s educational status	No formal education	524 (31.8)
	Primary school	642 (39)
	Secondary school Above secondary school	372 (22.6) 108 (6.6)
Woman’s occupation	Farmer	302 (18)
	Housewife	980 (60)
	Self-employed	211 (13)
	Day labourer	40 (2)
	Governmental	113 (7)
Income per month	<499.00Ebirr	159 (10)
	500–999.00Ebirr	297 (18)
	1000–1499.00Ebirr	549 (33)
	>1500.00Ebirr	641 (39)
Husband’s education	No formal education	449 (27.3)
	Completed primary school	493 (30)
	Completed secondary school	419 (26)
	Above secondary school	285 (17)
Husband’s occupation	Unemployed	73(4.4)
	Farmer	578 (35.3)
	Self-employed/merchant	465 (28.4)
	Daily laborer	234 (14.3)
	Government employee	296 (18)
Household Media	None	929 (56.4)
	Yes	717 (43.6)
Media source (among those with household media)	Radio	299 (42.7)
	Television	233 (33.2)
	Radio and television	169 (24.1)

### Obstetric history related factors

The majority of women (n = 1,165, 70.9%) were multigravida and 713 (43.3%) had three or more children. Approximately one-third of participants (n = 611) had their first pregnancy before age 19 years. Pregnancy loss was reported by 11% of participants, 84 (5.1%) of women reported a history of neonatal death and 100 (6.0%) women reported a history of stillbirth. Nearly three-quarters of women (n = 1261) gave birth in a health facility. More than 88% (n = 1458) had antenatal care (ANC) and 1,167 (70.9%) had both ANC and Tetanus toxoid vaccination. Most pregnancies were planned or desired (80.6%) (Table 2).

**Table 2:** Obstetric history related factors among postnatal women in Tigray, Ethiopia 2017(n = 1646).

Variables	Category	Frequency n (%)
Age at first pregnancy		
	≤18 & 19 yrs.	611 (37.1)
	≥20yrs.	1035 (62.9)
History of abortion	No	1429 (86.8)
	Yes	217 (13.2)
History of stillbirth	No	1546 (93.9)
	Yes	100 (6.1)
History of Infant death	No	1562 (94.9)
	Yes	84 (5.1)
Number of live children	1–2 children	956 (58.1)
	3-4children	491 (29.8)
	5+ children	199 (12.1)
Was the pregnancy wanted?	Wanted	1326 (80.6)
	Unwanted	320 (19.4)
Antenatal care frequency	Two	144 (9.8)
	Three	497 (34)
	Four+	824 (56.2)
TT frequency	One	302 (25.9)
	Two	539 (46.2)
	Three and above	326 (27.9)
Mode of delivery**	Vaginal birth (home) Vaginal birth (hospital)	385 (23.4) 1100 (66.8)
	Cesarean	99 (6.0)
	Vacuum or forceps	62 (3.8)
Pre-lactal feeding	Colostrum	1417 (86.1)
	Butter	174 (10.6)
	Wet nursing	55 (3.3)
Breastfeeding	Did not breastfeed	87 (5.3)
	Within 1 hr.	1033 (62.8)
	Within 1–24 hr.	411 (25.0)
	After one day	115 (6.9)
Baby vaccine	No	72 (4.4)
	Yes	1574 (95.6)

### Knowledge of maternal and infant danger signs

Participants were queried regarding their knowledge of potential postnatal danger signs. Participants were able to select multiple answers as part of the query. More than half of the participants (55.6%) knew at least one postnatal maternal danger sign such as bleeding too much, and similarly the majority (55.7%) knew at least one infant danger sign such as inability to breastfeed or vomiting. Fewer women knew of the benefits of postnatal care service (30.7%), and of those that knew the benefits, 254 (50.2%) said postnatal follow-up is used to get both immunizations and for family planning (Table 3).

**Table 3:** Mothers’ knowledge related to danger signs and benefits of postnatal care.

Variables	Category	Frequency (%)
Infant danger sign	Don’t know any	746 (45.3)
	Know at least 1 danger sign	900 (55.7)
Maternal danger signs	Don’t know any	731 (44.4)
	Know at least 1 danger sign	915 (55.6)
Ever heard of postnatal care?	No	1376 (83.6)
	Yes	270 (16.4)
Source of postnatal care information	From healthcare provider	243 (90)
	From family or neighbor	27 (10%)
Does postnatal care have benefits?	No	1140 (69.3)
	Yes	506 (30.7)
Benefits of postnatal care identified by participants	To prevent health problems of the mother	63 (30.7)
	To prevent any health problems of the baby	83 (16.4)
	To prevent both health problems of the mother and baby	106 (20.9)
	Counseling on immunization and family planning	254 (50.2)

### Postnatal care related factors for mothers who received postnatal care

Only 132 (8%) of the mothers reported receiving any postnatal care. Among those who attended postnatal care, 101 (76.5%) knew the importance of postnatal care. Most (81.8%) of the mothers’ mode of transportation was by foot. A significant number of women (40.2%) who attended postnatal care reported that waiting was a problem when obtaining postnatal care, however most (74.2%) of the mothers reported waited less than one hour to get the service (Table 4).

**Table 4:** Postnatal care related factors among mothers who attended postnatal care women in Tigray, Ethiopia 2017(n = 132).

Variables	Category	Frequency n (%)
Attended postnatal care at 6 weeks post childbirth	No	1514 (92)
	Yes	132 (8)
Person(s) who made decision to seek postnatal care	Only mother	48 (36.6)
	Only husband	21 (16)
	Both mother and husband	38 (29)
	With other family member	24 (18.3)
The postnatal service the mothers were aware of	Physical examination	9 (8.9)
	Counseling and service on breastfeeding, family planning, and immunization	92 (91.1)
Waiting to get the service was problem	No	79 (59.8)
	Yes	53 (40.2)
How long mother waited to get postnatal care service once at facility	<1hr	98 (74.2)
	1-2hr	23 (17.4)
	>2hr	11 (8.3)
Transport to health facility	Foot	10(81.8)
	Public	24 (18.2)
Travel time to health facility	<1hr	116 (87.8)
	1-2hr	13 (9.8)
	>2hr	3 (2.3)
Cost of transport	No cost	73 (55.3)
	Less than or equal to 20 birr	43 (32.6)
	Greater than 20 birr	16 (12.1)
Accompanied by others to postnatal care	Only mother	30 (22.7)
	With husband	39 (29.5)
	With other family members	63 (47.7)

### Barriers for not attending postnatal care

Ninety two percent of mothers did not use postnatal care, with the majority (62.9%) of these women indicating that there were barriers to receiving postnatal care. The most common reason was that women were not told of the importance of postnatal care by healthcare providers. Many women (n = 211) indicated disrespectful maternity care (being examined roughly, being shouted at or ignored) as a reason for not returning for postnatal care (Table 5)

**Table 5:** Reasons for not attending postnatal care among women in Tigray, Ethiopia 2017 (n = 1514).

Variables	Category	Frequency n (%)
Are there barriers to getting postnatal care?	No	561 (37.1)
	Yes	953 (62.9)
If yes, what are they?	The health care providers did not tell me I had to return	742 (77.9)
	Examined me roughly	34 (3.6)
	Shouted at me and they did not teach me well	105 (11.5)
	The health care providers examined me roughly and ignored me	72 (7.6)
Did mother have cultural beliefs that hinder postnatal care?	No	1398 (92.3)
	Yes	116 (7.7)
Cultural reasons	Cannot go outside before 12 days—45 days	50 (43.1)
	Devil will get them on the way	12 (10.3)
	Michi*	54 (46.6)

* a lesion that is believed to occur as a result of sun exposure immediately after delivery.

### Factors associated with postnatal care service utilization

Demographic variables that were associated with use of postnatal care services included living in an urban area, higher educational status, being employed, and having a higher income. Of the obstetric variables, having more than 3 ANC visits, having a wanted pregnancy, giving birth in a health facility, delivering by cesarean section, increased the likelihood of attending postnatal care. Knowledge of postnatal care or knowledge of infant danger signs, exposure to media (listening to radio, watching TV, reading printed materials) were positively associated with postnatal care attendance. Marital status, age, religion and ethnicity were not associated with postnatal care attendance. Similarly, prior obstetric history (number of births, history of abortion or stillbirth) were not associated with postnatal care attendance (Table 6). Other variables were used in the analysis but only those with significant associations (variables with a P value, <0.05) in the bivariate analysis were included in the multivariate logistic regression.

**Table 6:** Association of factors with postnatal care service, Tigray region, Ethiopia (n = 1646).

Variable	Category n (%)	Crude odds ratio (COR) (95% CI)	Adjusted odds ratio (AOR) (95% CI)
Residence	Rural 1417 (86.1)	reference	reference
	Urban 229 (13.9)	3.59 (2.42–5.32)**	1.96 (1.07, 3.59) *
Woman’s educational status	No formal education 524 (31.8)Primary school 642 (39)	Reference1.49 (0.84,2.64)	reference 0.78 (0.37, 1.64)
	Secondary school 372 (22.6)	4.45 (2.57, 7.60) **	1.46 (0.68, 2.97)
	Above secondary school 108 (6.6)	8.43 (4.46, 15.9) **	3.60 (1.32, 9.83) *
Woman’s occupation	Farmer 302 (18)	reference	reference
	Housewife Self-employed 211 (13)	0.00 (0.00, 0.00) 2.99 (1.96, 4.58) **	0.00 (0.00, 0.00) 2.74 (1.41, 5.30)*
	Day labourer 40 (2)	2.50 (1.43, 4.38) *	0.40 (0.16, 1.00)
	Governmental 113 (7)	2.33 (0.95, 5.74)	2.95 (0.70, 12.5)
Any media exposure	No 929 (56.4)	reference	reference
	Yes 717 (43.6)	4.35 (2.90, 6.54) **	2.17 (1.21, 3.88) *
ANC frequency	Two 144 (9.8)	reference	reference
	Three 497 (34)	1.53 (0.63, 3.74)	0.99 (0.31, 3.19)
	Four+ 824 (56.2)	2.93 (1.26, 6.81) *	4.84 (1.57, 14.9)*
Was the pregnancy wanted?	Unwanted 320 (19.4)Wanted 1326 (80.6)	reference 8.44 (3.01, 23.0) **	6.47 (2.04, 20.5) *
Mode of Delivery	Vaginal 1485 (87.3)	reference	reference
	C/S 99 (7.8)	2.14 (1.22, 3.76) *	2.88 (1.32, 6.29) *
	Vacuum and forceps 62 (4.9)	2.48 (1.28, 4.82) *	2.57 (0.96, 6.87)
Mother’s perception of whether there are benefits of postnatal care	No 1140 (69)Yes 506 (30.7)	reference 7.33 (4.91, 11.0)**	reference 5.49 (3.06, 9.83) **
Knowledge of infant danger signs	Does not know 746 (45.3)	reference	reference
	Knows ≥1 danger sign 900 (455.7)	2.18 (1.18, 4.02) *	3.59 (1.57, 8.18) *

* p value <0.025

** p value < 0.001

## Discussion

Among women in the Tigray region of Ethiopia, only 8% received postnatal care within six weeks of delivery. Women were more likely to receive postnatal services if they knew about the services, lived in an urban area, had greater than a secondary education, had a cesarean birth, had at least four antenatal visits, and if the pregnancy was wanted. Our findings show an even lower proportion of women obtained postnatal care compared to previous studies done in Ethiopia. Estimates of postnatal care following delivery in prior studies range from 20.2% to 66.8% [8,17–20,21]. A possible explanation for the low attendance at postnatal care in Tigray could be that we only considered postnatal services given by skilled health professionals, whereas the comparative studies considered care given both by health extension workers and healthcare professionals. Additionally, it could be that in Tigray there is less value placed on postnatal care by the women, families and their healthcare providers.

About a quarter (385) of participants delivered at home. This is lower than previous studies done in other parts of Ethiopia. In West Shewa, Oromiya Region 85.6% reported a home delivery [18], while in Northwest Ethiopia 68.6% of women had home deliveries [20]. This decrease in births occurring outside the health facility could be due to recent efforts to use frontline extension workers to educate communities on the availability of health services provided by the government. However, there is question on if the same outreach is emphasizing the importance of postnatal care. In addition, in Tigray there is organized community health support women developmental army (representative of women who works mobilizing health of the community) and the linkage between this group and health extension to link the women to health facility delivery.

Approximately 90% of the mothers in our study received ANC services during their pregnancy, which is higher compared with a study done in Northwest Ethiopia which revealed that 76.6% of women received ANC service during their last pregnancy [20]. The possible explanation for this could be due to the well-organized women developmental army that works in health promotion in Tigray. What is concerning, is that despite the higher percentage of women attending antenatal care, so few women returned for postnatal care, suggesting that not enough emphasis is being placed on the importance of postnatal care. Mothers who attended four or more ANC visits were more likely to attend postnatal care, results similar to those reported in a study of postnatal care attendance in Amhara Region, Ethiopia[22]. Additionally, use of postnatal care was higher among women who had experienced problems during their delivery and/or mothers who had a cesarean birth, similar to a prior study in Northwestern Ethiopia [23].

The pattern of postnatal care usage is consistent with previous studies. Of all the participants who attended postnatal care, about 38% of them attended within 4–24 hours after delivery. This is similar to a study in Northwest Ethiopia where 34% of women who attended postnatal care visits attended early postnatal care [20]. Similar to other studies in both Ethiopia [20] and other countries [24], many women did not utilize postnatal services because they were not aware that the services existed, nor did they know the benefits.

Cultural practices have been associated with non-utilization of postnatal care in studies conducted in rural, China [24] and Bangladesh [25]. In this study only a small percentage (7.7%) of women indicated cultural beliefs such as postnatal mothers do not go outside before 12–45 days as a barrier to seeking postnatal care.

Socioeconomic factors influenced the likelihood of using postnatal care services. The mothers who completed at least a secondary education were more likely to utilize postnatal care services then women who had not. This is similar to findings of a study done in Jabitena District in Ethiopia [17]. Employment in mothers was significantly associated with postnatal care service utilization. The finding is similar to the findings in Northwest Ethiopia [17) and Nepal [24]. Women were also more likely to receive postnatal services if they lived in an urban area; consistent with a study in Ethiopia’s Amhara region, where urban residence was associated with postnatal care utilization [20]. Similar to findings of a study done in the Jabitena district, Amhara region, Ethiopia [17], we found that education about postpartum health or the benefits of postnatal care impacted use of services.

A significant and concerning finding was that a number of women reported disrespectful maternity care, being treated roughly or being shouted as a reason for not returning for postnatal care. Respectful maternity care is not only a basic human right, when healthcare providers are disrespectful it can result in sub-optimal healthcare utilization [25].

## Limitations

The largest limitation of this study is that it was a cross-sectional study. The primary limitation of the cross-sectional study design is that because the exposure and outcome are simultaneously assessed, there is generally no evidence of a temporal relationship between exposure and outcome. Additionally, this study relied on self-report of the women rather than healthcare records of obstetric variables and postnatal care attendance. Lastly, participants were from one area in Ethiopia, therefore the findings from this study are not generalizable to other parts of Ethiopia or sub-Saharan Africa.

## Conclusion and recommendation

Although the care given during the postpartum period is very important in preventing maternal and neonatal morbidity and mortality, postnatal care service utilization is very low in the Tigray region. Targeted interventions seeking to address this concern should be multifaceted and include both improving awareness about the need for postnatal care service and training of healthcare providers to provide respectful, quality care during pregnancy, labor, birth and beyond so that women are not deterred from seeking care during the postnatal period. Endeavors to increase awareness of the importance of postnatal care should be targeted not only to women, but also families and healthcare providers. Awareness should include information and education provided during prenatal care and prior to discharge following birth. More emphasis should be placed on the importance, as well as the components, of postnatal care in pre-service healthcare educational programs and continuing education with midwives, nurses and physicians. Further research should include the perspective of health care providers regarding the barriers to providing postnatal care to mothers. In addition, health extensions workers should focus educational efforts on the importance of care given during the postnatal period as well as during the pregnancy and birth.

## References

[1] Trends in maternal mortality: 1990 to 2013 Estimates by WHO, UNICEF, UNFPA, The World Bank and the United Nations Population Division. Geneva: World Health Organization; 2014.

[2] Trends in maternal mortality: 1990 to 2010 WHO, UNICEF, UNFPA and The World Bank Estimates. Geneva: World Health Organization; 2012.

[3] Lawn J, Blencowe H, Oza S, You D, Lee A, Waiswa P, et al Every Newborn: progress, priorities, and potential beyond survival. Lancet 2014; 384: 189–205. 10.1016/S0140-6736(14)60496-7 24853593

[4] Ronsmans C, Graham W, Lancet Maternal Survival Series steering group. Maternal mortality: who, when, where, and why. Lancet. 2006; 368: 1189–200. 10.1016/S0140-6736(06)69380-X 17011946

[5] Sines E, Syed U, Wall S, Worley H. "Postnatal care: A critical opportunity to save mothers and newborns. 2007." Population Reference Bureau, Washington DC Google Scholar 2015.

[6] Rahman M, Haque S, Zahan M. Factors affecting the utilization of postpartum care among young mothers in Bangladesh. Health Soc Care Community. 2011;19: 138–47. 10.1111/j.1365-2524.2010.00953.x 20880103

[7] Afework M. Achieving the maternal health Millennium Development Goals in Ethiopia: Where we are and what needs to be done? Ethiopian Journal of Health Development. 2010; 24: 87–88.

[8] Ethiopia demographic and health survey 2016. Accessed June 11, 2018 https://dhsprogram.com/pubs/pdf/fr255/fr255.pdf

[9] Gabrysch S, Campbell O. Still too far to walk: literature review of the determinants of delivery service use. BMC Pregnancy Childbirth. 2009; 9:34 10.1186/1471-2393-9-34 19671156 PMC2744662

[10] Say L, Raine R. A systematic review of inequalities in the use of maternal health care in developing countries: examining the scale of the problem and the importance of context. Bull World Health Organ. 2007; 85: 812–9. 10.2471/BLT.06.035659 10.2471/BLT.06.035659 . 18038064 PMC2636485

[11] WHO recommendations on postnatal care of the mother and newborn. Geneva: World Health Organization; 2014. 24624481

[12] Matijasevich A, Santos I, Silveira M, Domingues M, Barros A, Marco P, et al Inequities in maternal postnatal visits among public and private patients: 2004 Pelotas cohort study. BMC Public Health. 2009; 9: 335 10.1186/1471-2458-9-335 19751521 PMC2749044

[13] “Population and housing census -2007”(PDF). FDRE Population Census Commission Retrieved 6 October 2016.

[14] Tigray regional health bureau annual profile 2007 Ethiopian fiscal year.

[15] Workineh Y. Factors affecting utilization of postnatal care service in Jabitena district, Amhara region, Ethiopia. Sci J Public Health. 2014; 23: 169–76

[16] Medécins Sans Frontières: Refugee Health: An Approach to Emergency Situations London: MacMillian Education Ltd; 1997

[17] Expanded Programme on Immunization: Immunization coverage cluster survey–Reference manual: WHO/IVB/04.23. [http://www.who.int/vaccinesdocuments/DocsPDF05/www767.pdf] https://www.researchgate.net/publication/6293463_Don%27t_spin_the_pen_Two_alternative_methods_for_second-stage_sampling_in_urban_cluster_surveys [accessed Nov 11 2017].

[18] Darega B, Tafese F, Ololo S. 2016 Jul 7; Institutional delivery and postnatal care services utilizations in Abuna Gindeberet District, West Shewa, Oromiya Region, Central Ethiopia: A Community-based cross-sectional study. BMC pregnancy and childbirth. 2016; 16:149 10.1186/s12884-016-0940-x 27386945 PMC4936291

[19] Regassa N. Antenatal and postnatal care service utilization in southern Ethiopia: a population-based study. African health sciences. 2011: 11; 390–397. 22275929 PMC3260999

[20] Tesfahun F, Mazengiya F, Kifle M. Knowledge, perception and utilization of postnatal care of mothers in Gondar Zuria District, Ethiopia: a cross-sectional study. Maternal and child health journal. 2014; 10: 2341–51. 10.1007/s10995-014-1474-3 PMC4220106 24770953

[21] Limenih M, Dachew B. Postnatal Care Service Utilization and Associated Factors among Women Who Gave Birth in the Last 12 Months prior to the Study in Debre Markos Town, Northwestern Ethiopia: A Community-Based Cross-Sectional Study. International journal of reproductive medicine. 2016; 10: 1–7. 10.1155/2016/7095352 PMC4940555 27433481

[22] Khanal V. Factors associated with the utilization of postnatal care services among the mothers of Nepal: analysis of Nepal Demographic and Health Survey 2011. BMC Women's Health. 2014; 14: 19 10.1186/1472-6874-14-19 24484933 PMC3911793

[23] Chen L. Coverage, quality of and barriers to postnatal care in rural Hebei, China: a mixed method study. BMC Pregnancy and Childbirth. 2014; 14: 31 10.1186/1471-2393-14-31 24438644 PMC3898028

[24] Winch P, Akther A, Afroz D, Ali N, Ellis A, Baqui A, et al Local understandings of vulnerability and protection during the neonatal period in Sylhet district, Bangladesh: a qualitative study. Lancet. 2005; 366: 478–485. 10.1016/S0140-6736(05)66836-5 16084256

[25] Miller S, Abalos E, Chamillard M, Ciapponi A, Colaci D, Comande D, et al Beyond too little, too late and too much, too soon: a pathway towards evidence-based, respectful maternity care worldwide. Lancet 2016; 388; 2176–219 10.1016/S0140-6736(16)31472-6 27642019

